# 9-Ethyl-10-methyl­acridinium trifluoro­methane­sulfonate

**DOI:** 10.1107/S1600536808039676

**Published:** 2008-12-06

**Authors:** Beata Zadykowicz, Michał Wera, Artur Sikorski, Jerzy Błażejowski

**Affiliations:** a Faculty of Chemistry, University of Gdańsk, Sobieskiego 18, 80-952 Gdańsk, Poland

## Abstract

In the mol­ecule of the title compound, C_16_H_16_N^+^·CF_3_SO_3_^−^, the central ring adopts a flattened-boat conformation, and the two aromatic rings are oriented at a dihedral angle of 3.94 (2)°. In the crystal structure, weak inter­molecular hydrogen bonds link the mol­ecules. There are π–π contacts between the aromatic rings and the central ring and one of the aromatic rings [centroid–centroid distances = 3.874 (2), 3.945 (2) and 3.814 (2) Å]. There is also an S—O⋯π contact between the central ring and one of the O atoms of the anion.

## Related literature

For general background, see: Bianchi *et al.* (2004 ▶); Dorn *et al.* (2005 ▶); Hunter & Sanders (1990 ▶); Steiner (1991 ▶); Suzuki & Tanaka (2001 ▶); Zomer & Jacquemijns (2001 ▶). For related structures, see: Huta *et al.* (2002 ▶); Krzymiński *et al.* (2007 ▶); Meszko *et al.* (2002 ▶); Sikorski *et al.* (2005*a* ▶,*b* ▶,*c* ▶, 2006 ▶, 2008 ▶); Storoniak *et al.* (2000 ▶); Tsuge *et al.* (1965 ▶). For ring puckering parameters, see: Cremer & Pople (1975 ▶).
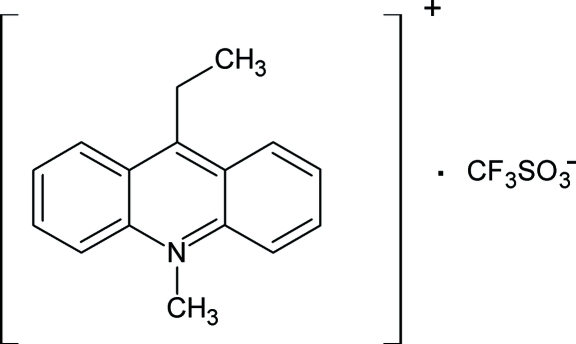


## Experimental

### 

#### Crystal data

C_16_H_16_N^+^·CF_3_SO_3_^−^
*M* *_r_* = 371.37
Triclinic, 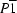

*a* = 7.771 (2) Å
*b* = 9.440 (2) Å
*c* = 11.898 (2) Å
α = 76.76 (3)°
β = 74.04 (3)°
γ = 82.14 (3)°
*V* = 814.3 (3) Å^3^
*Z* = 2
Mo *K*α radiation
μ = 0.25 mm^−1^
*T* = 295 (2) K
0.5 × 0.5 × 0.05 mm

#### Data collection

Oxford Diffraction GEMINI R ULTRA Ruby CCD diffractometer
Absorption correction: multi-scan (*CrysAlis RED*; Oxford Diffraction, 2008 ▶) *T*_min_ = 0.870, *T*_max_ = 0.988
7781 measured reflections
2857 independent reflections
2078 reflections with *I* > 2σ(*I*)
*R*_int_ = 0.020

#### Refinement

*R*[*F* ^2^ > 2σ(*F* ^2^)] = 0.035
*wR*(*F* ^2^) = 0.102
*S* = 1.08
2857 reflections
229 parameters
H-atom parameters constrained
Δρ_max_ = 0.21 e Å^−3^
Δρ_min_ = −0.25 e Å^−3^

### 

Data collection: *CrysAlis CCD* (Oxford Diffraction, 2008 ▶); cell refinement: *CrysAlis RED* (Oxford Diffraction, 2008 ▶); data reduction: *CrysAlis RED*; program(s) used to solve structure: *SHELXS97* (Sheldrick, 2008 ▶); program(s) used to refine structure: *SHELXL97* (Sheldrick, 2008 ▶); molecular graphics: *ORTEPII* (Johnson, 1976 ▶); software used to prepare material for publication: *SHELXL97* and *PLATON* (Spek, 2003 ▶).

## Supplementary Material

Crystal structure: contains datablocks global, I. DOI: 10.1107/S1600536808039676/hk2577sup1.cif

Structure factors: contains datablocks I. DOI: 10.1107/S1600536808039676/hk2577Isup2.hkl

Additional supplementary materials:  crystallographic information; 3D view; checkCIF report

## Figures and Tables

**Table 1:** Hydrogen-bond geometry (Å, °)

*D*—H⋯*A*	*D*—H	H⋯*A*	*D*⋯*A*	*D*—H⋯*A*
C2—H2⋯O23^i^	0.93	2.47	3.369 (3)	164
C15—H15*C*⋯O24^ii^	0.96	2.40	3.276 (3)	151
C16—H16*B*⋯O25^iii^	0.97	2.58	3.377 (3)	140

Symmetry codes: (i) 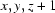; (ii) 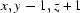; (iii) 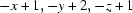.

**Table 2:** π–π Interactions (Å, °)

*CgI*	*CgJ*	*Cg*⋯*Cg*	Dihedral angle	Interplanar distance	Offset
1	2^iv^	3.814 (2)	3.88	3.517 (2)	5.188
2	1^iv^	3.814 (2)	3.88	3.542 (2)	5.205
2	2^iv^	3.945 (2)	0.02	3.578 (2)	5.326
2	2^v^	3.874 (2)	0.02	3.440 (2)	5.181

Symmetry codes: (iv) 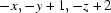; (v) 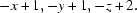 *Cg*1 and *Cg*2 are the centroids of the C9/N10/C11–C14 and C1–C4/C11/C12 rings, respectively. *Cg*⋯*Cg* is the distance between ring centroids. The dihedral angle is that between the planes of the rings *CgI* and *CgJ*. The interplanar distance is the perpendicular distance of *CgI* from ring *J*. The offset is the perpendicular distance of ring *I* from ring *J*.

**Table 3:** S—O⋯π Interactions (Å, °)

*X*	*I*	*J*	*I*⋯*J*	*X*⋯*J*	*X*—*I*⋯*J*
S22	O23	*Cg*1^vi^	3.255 (2)	3.072 (2)	146

Symmetry codes: (vi) 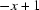, 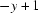, 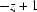. *Cg*1 is the centroid of the C9/N10/C11–C14 ring.

**Fig. 1.**
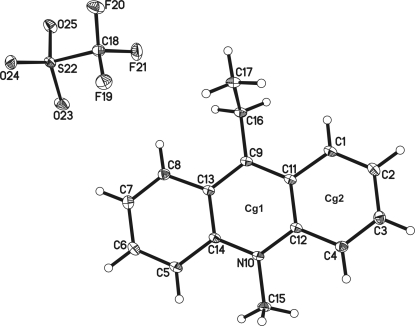
The molecular structure of the title compound showing the atom-labeling scheme. Displacement ellipsoids are drawn at the 25% probability level and H atoms are shown as small spheres of arbitrary radius. Cg1 and Cg2 denote the ring centroids.

**Fig. 2.**
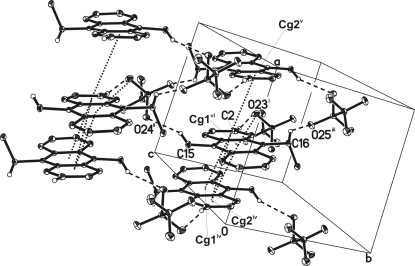
The arrangement of the ions in the crystal structure. The C—H···O interactions are represented by dashed lines, the π-π and S—O···π interactions by dotted lines. H atoms not involved in the interactions have been omitted. [Symmetry codes: (i) x, y, z + 1; (ii) x, y - 1, z + 1; (iii) -x, -y + 2, -z + 1; (iv) -x, -y + 1, -z + 2; (v) -x + 1, -y + 1, -z + 2; (vi) -x + 1, -y + 1, -z + 1.]

## supplementary crystallographic information

### Comment

Acridinium cations substituted in positions 9 and 10 are susceptible to attack by OOH^-^ or other oxidants at C9, which initiates conversion of these cations to electronically excited-light emitting 9-acridinones (Zomer & Jacquemijns, 2001). We investigated the above described chemiluminescence in the case of 9-(phenoxycarbonyl)-10-methylacridinium trifluoromethanesulfonates, the several structures of which we recently determined (Sikorski *et al.*, 2005*a*, b, c; Sikorski *et al.*,  2006; Krzymiński *et al.*, 2007; Sikorski *et al.*,  2008). Chemiluminogenic features are also exhibited by the 9-cyano-10-methylacridinium and 9,10-dimethylacridinium cations respectively present as counterpart ions in hydrogen dinitrate and methylsulfate salts, the crystal structures of which were also refined (Huta *et al.*,  2002; Meszko *et al.*, 2002). We report herein the crystal structure of the title compound, which was selected for investigations as a potential chemiluminogen. The 9-ethyl-10-methylacridinium cation may also be interesting as a model compound in investigations of C-acidic features of organic molecules, since such properties are exhibited by the 9,10-dimethylacridinium cation (Suzuki & Tanaka, 2001).

In the molecule of the title compound (Fig. 1) the bond lengths and angles, characterizing the geometry of the acridine ring, are typical of acridine-based derivatives (Storoniak *et al.*,  2000; Meszko *et al.*, 2002). Rings A (C1-C4/C11/C12) and C (C5-C8/C13/C14) are planar and are oriented at a dihedral angle of 3.94 (2)°. Ring B (C9/N10/C11-C14) is not planar, having total puckering amplitude, Q_T_, of 1.990 (5) and flattened-boat conformation [φ = 31.52 (5)° and θ = 21.87 (4)°] (Cremer & Pople, 1975).

In the crystal structure, weak intermolecular hydrogen bonds (Table 1) link the molecules. The central ring B and the aromatic ring A are involved in multidirectional π-π interactions (Table 2, Fig. 2). One of the O atoms of the anion is involved in weak S—O···π interactions directed toward the center of the acridine ring system (Table 3, Fig. 2). The C—H···O (Bianchi *et al.*,  2004; Steiner, 1999) interactions are of the hydrogen-bond type. The S—O···π interactions (Dorn *et al.*,  2005) should be of an attractive nature, such as is also exhibited by π-π interactions (Hunter & Sanders, 1990). The crystal structure is stabilized by a network of the aforementioned short-range interactions, as well as by long-range electrostatic interactions between ions.

### Experimental

9-Ethylacridine was synthesized by heating a mixture of *N*-phenylaniline with an equimolar amount of propanoic acid, both dispersed in molten zinc chloride (493 K, 26 h) (Tsuge *et al.*, 1965). The crude product was purified by gravitational column chromatography (SiO_2_, n-hexane-ethyl acetate, 5:1 *v*/*v*). 9-Ethyl-10-methylacridinium trifluoromethanesulfonate was obtained by dissolving 9-ethylacridine with a fivefold molar excess of methyl trifluoromethanesulfonate in anhydrous dichloromethane and leaving the mixture for 3 h (Ar atmosphere, room temperature). The crude salt that precipitated was dissolved in a small amount of ethanol, filtered, and again precipitated with a 25 *v*/*v* excess of diethyl ether (yield; 89%). Pale-yellow crystals suitable for X-ray analysis were grown from absolute ethanol solution.

### Refinement

H atoms were positioned geometrically, with C-H = 0.93, 0.97 and 0.96 Å for aromatic, methylene and methyl H, respectively, and constrained to ride on their parent atoms with U_iso_(H) = xU_eq_(C), where x = 1.5 for methyl H and x = 1.2 for all other H atoms.

### Crystal data

**Table:**

C_16_H_16_N^+^·CF_3_SO_3_^−^	*Z* = 2
*M**_r_* = 371.37	*F*(000) = 384
Triclinic, *P*1	*D*_x_ = 1.515 Mg m^−^^3^
Hall symbol: -P 1	Mo *K*α radiation, λ = 0.71073 Å
*a* = 7.771 (2) Å	Cell parameters from 2857 reflections
*b* = 9.440 (2) Å	θ = 3.1–25.0°
*c* = 11.898 (2) Å	µ = 0.25 mm^−^^1^
α = 76.76 (3)°	*T* = 295 K
β = 74.04 (3)°	Plate, pale-yellow
γ = 82.14 (3)°	0.5 × 0.5 × 0.05 mm
*V* = 814.3 (3) Å^3^	

### Data collection

**Table:**

Oxford Diffraction GEMINI R ULTRA Ruby CCD diffractometer	2857 independent reflections
Radiation source: Enhance (Mo) X-ray Source	2078 reflections with *I* > 2σ(*I*)
graphite	*R*_int_ = 0.020
Detector resolution: 10.4002 pixels mm^-1^	θ_max_ = 25.0°, θ_min_ = 3.1°
ω scans	*h* = −9→8
Absorption correction: multi-scan (*CrysAlis RED*; Oxford Diffraction, 2008)	*k* = −8→11
*T*_min_ = 0.870, *T*_max_ = 0.988	*l* = −13→14
7781 measured reflections	

### Refinement

**Table:**

Refinement on *F*^2^	Secondary atom site location: difference Fourier map
Least-squares matrix: full	Hydrogen site location: inferred from neighbouring sites
*R*[*F*^2^ > 2σ(*F*^2^)] = 0.035	H-atom parameters constrained
*wR*(*F*^2^) = 0.102	*w* = 1/[σ^2^(*F*_o_^2^) + (0.0604*P*)^2^ + 0.0136*P*] where *P* = (*F*_o_^2^ + 2*F*_c_^2^)/3
*S* = 1.08	(Δ/σ)_max_ < 0.001
2857 reflections	Δρ_max_ = 0.22 e Å^−^^3^
229 parameters	Δρ_min_ = −0.25 e Å^−^^3^
0 restraints	Extinction correction: *SHELXL97* (Sheldrick, 2008), Fc^*^=kFc[1+0.001xFc^2^λ^3^/sin(2θ)]^-1/4^
Primary atom site location: structure-invariant direct methods	Extinction coefficient: 0.014 (3)

### Special details

**Table:**

Experimental. Empirical absorption correction using spherical harmonics, implemented in SCALE3 ABSPACK scaling algorithm.
Geometry. All e.s.d.'s (except the e.s.d. in the dihedral angle between two l.s. planes) are estimated using the full covariance matrix. The cell e.s.d.'s are taken into account individually in the estimation of e.s.d.'s in distances, angles and torsion angles; correlations between e.s.d.'s in cell parameters are only used when they are defined by crystal symmetry. An approximate (isotropic) treatment of cell e.s.d.'s is used for estimating e.s.d.'s involving l.s. planes.
Refinement. Refinement of *F*^2^ against ALL reflections. The weighted *R*-factor *wR* and goodness of fit *S* are based on *F*^2^, conventional *R*-factors *R* are based on *F*, with *F* set to zero for negative *F*^2^. The threshold expression of *F*^2^ > σ(*F*^2^) is used only for calculating *R*-factors(gt) *etc*. and is not relevant to the choice of reflections for refinement. *R*-factors based on *F*^2^ are statistically about twice as large as those based on *F*, and *R*- factors based on ALL data will be even larger.

### Fractional atomic coordinates and isotropic or equivalent isotropic displacement parameters (Å^2^)

**Table:**

	*x*	*y*	*z*	*U*_iso_*/*U*_eq_	
C1	0.3057 (2)	0.6242 (2)	0.97192 (18)	0.0479 (5)	
H1	0.3455	0.7172	0.9413	0.057*	
C2	0.2727 (3)	0.5708 (2)	1.09064 (19)	0.0541 (5)	
H2	0.2876	0.6276	1.1411	0.065*	
C3	0.2161 (3)	0.4299 (2)	1.13769 (19)	0.0551 (5)	
H3	0.1946	0.3940	1.2195	0.066*	
C4	0.1918 (3)	0.3445 (2)	1.06641 (18)	0.0490 (5)	
H4	0.1571	0.2503	1.0993	0.059*	
C5	0.1504 (3)	0.2917 (2)	0.68005 (19)	0.0526 (5)	
H5	0.1046	0.2013	0.7141	0.063*	
C6	0.1686 (3)	0.3478 (2)	0.5618 (2)	0.0616 (6)	
H6	0.1340	0.2947	0.5159	0.074*	
C7	0.2377 (3)	0.4825 (2)	0.50750 (19)	0.0594 (6)	
H7	0.2505	0.5175	0.4262	0.071*	
C8	0.2859 (3)	0.5621 (2)	0.57323 (17)	0.0509 (5)	
H8	0.3312	0.6522	0.5364	0.061*	
C9	0.3131 (2)	0.59454 (18)	0.76881 (17)	0.0397 (4)	
N10	0.18592 (19)	0.31700 (15)	0.86993 (13)	0.0387 (4)	
C11	0.2806 (2)	0.54049 (18)	0.89252 (17)	0.0394 (4)	
C12	0.2190 (2)	0.39871 (18)	0.94283 (16)	0.0388 (4)	
C13	0.2688 (2)	0.51098 (19)	0.69798 (16)	0.0398 (4)	
C14	0.2018 (2)	0.37195 (19)	0.75118 (17)	0.0397 (4)	
C15	0.1358 (3)	0.1658 (2)	0.92047 (19)	0.0548 (5)	
H15A	0.1862	0.1064	0.8619	0.082*	
H15B	0.0074	0.1650	0.9426	0.082*	
H15C	0.1813	0.1277	0.9897	0.082*	
C16	0.3835 (3)	0.7413 (2)	0.71458 (19)	0.0492 (5)	
H16A	0.4536	0.7416	0.6333	0.059*	
H16B	0.4621	0.7601	0.7594	0.059*	
C17	0.2316 (3)	0.8623 (2)	0.7141 (2)	0.0590 (6)	
H17A	0.2808	0.9541	0.6753	0.088*	
H17B	0.1670	0.8665	0.7948	0.088*	
H17C	0.1515	0.8423	0.6720	0.088*	
C18	0.2605 (3)	0.9709 (2)	0.36020 (18)	0.0563 (5)	
F19	0.12725 (18)	0.89034 (15)	0.42780 (11)	0.0835 (4)	
F20	0.19886 (19)	1.11050 (15)	0.35499 (12)	0.0838 (4)	
F21	0.3880 (2)	0.94796 (18)	0.41894 (12)	0.0913 (5)	
S22	0.34301 (7)	0.92827 (5)	0.21309 (4)	0.0488 (2)	
O23	0.4120 (2)	0.77973 (17)	0.23799 (17)	0.0817 (5)	
O24	0.18424 (19)	0.95139 (17)	0.17045 (13)	0.0650 (4)	
O25	0.47217 (19)	1.03268 (17)	0.15403 (13)	0.0688 (5)	

### Atomic displacement parameters (Å^2^)

**Table:**

	*U*^11^	*U*^22^	*U*^33^	*U*^12^	*U*^13^	*U*^23^
C1	0.0441 (11)	0.0382 (11)	0.0692 (14)	−0.0014 (8)	−0.0219 (10)	−0.0183 (10)
C2	0.0527 (13)	0.0543 (13)	0.0663 (14)	0.0040 (10)	−0.0248 (11)	−0.0271 (11)
C3	0.0548 (13)	0.0607 (14)	0.0535 (12)	0.0015 (10)	−0.0206 (10)	−0.0138 (10)
C4	0.0473 (12)	0.0422 (11)	0.0587 (13)	−0.0025 (9)	−0.0182 (9)	−0.0072 (9)
C5	0.0559 (13)	0.0417 (12)	0.0660 (14)	−0.0073 (9)	−0.0171 (10)	−0.0182 (10)
C6	0.0667 (15)	0.0633 (15)	0.0669 (15)	−0.0079 (12)	−0.0235 (12)	−0.0275 (12)
C7	0.0609 (14)	0.0682 (15)	0.0523 (12)	−0.0046 (11)	−0.0177 (11)	−0.0147 (11)
C8	0.0478 (12)	0.0491 (12)	0.0555 (13)	−0.0064 (9)	−0.0130 (10)	−0.0083 (10)
C9	0.0296 (10)	0.0320 (10)	0.0584 (12)	−0.0004 (7)	−0.0121 (8)	−0.0107 (8)
N10	0.0355 (8)	0.0285 (8)	0.0541 (10)	−0.0026 (6)	−0.0129 (7)	−0.0103 (7)
C11	0.0288 (9)	0.0336 (10)	0.0600 (12)	0.0019 (7)	−0.0154 (8)	−0.0155 (8)
C12	0.0305 (9)	0.0329 (10)	0.0557 (12)	0.0018 (7)	−0.0145 (8)	−0.0124 (8)
C13	0.0318 (10)	0.0346 (10)	0.0540 (11)	−0.0005 (7)	−0.0108 (8)	−0.0124 (8)
C14	0.0330 (10)	0.0340 (10)	0.0547 (12)	0.0009 (7)	−0.0125 (8)	−0.0146 (8)
C15	0.0676 (14)	0.0329 (11)	0.0676 (13)	−0.0116 (10)	−0.0219 (11)	−0.0070 (9)
C16	0.0468 (12)	0.0394 (11)	0.0621 (12)	−0.0107 (9)	−0.0124 (9)	−0.0093 (9)
C17	0.0638 (14)	0.0367 (12)	0.0740 (14)	−0.0036 (10)	−0.0152 (11)	−0.0091 (10)
C18	0.0564 (13)	0.0557 (14)	0.0565 (13)	−0.0210 (11)	−0.0115 (11)	−0.0041 (10)
F19	0.0803 (10)	0.0913 (11)	0.0695 (9)	−0.0423 (8)	0.0020 (7)	−0.0019 (7)
F20	0.0990 (11)	0.0619 (9)	0.0841 (9)	−0.0101 (8)	0.0033 (8)	−0.0317 (7)
F21	0.0951 (11)	0.1268 (13)	0.0649 (9)	−0.0367 (10)	−0.0363 (8)	−0.0091 (8)
S22	0.0461 (3)	0.0466 (3)	0.0603 (3)	−0.0064 (2)	−0.0172 (2)	−0.0179 (2)
O23	0.0841 (12)	0.0528 (10)	0.1235 (14)	0.0168 (8)	−0.0476 (11)	−0.0355 (9)
O24	0.0598 (9)	0.0738 (10)	0.0708 (9)	−0.0122 (8)	−0.0342 (8)	−0.0078 (8)
O25	0.0612 (10)	0.0840 (11)	0.0630 (9)	−0.0324 (8)	0.0022 (7)	−0.0248 (8)

### Geometric parameters (Å, °)

**Table:**

C1—C2	1.351 (3)	N10—C14	1.366 (2)
C1—C11	1.428 (2)	N10—C12	1.377 (2)
C1—H1	0.9300	N10—C15	1.477 (2)
C2—C3	1.399 (3)	C11—C12	1.424 (2)
C2—H2	0.9300	C13—C14	1.421 (3)
C3—C4	1.359 (3)	C15—H15A	0.9600
C3—H3	0.9300	C15—H15B	0.9600
C4—C12	1.408 (3)	C15—H15C	0.9600
C4—H4	0.9300	C16—C17	1.526 (3)
C5—C6	1.360 (3)	C16—H16A	0.9700
C5—C14	1.418 (3)	C16—H16B	0.9700
C5—H5	0.9300	C17—H17A	0.9600
C6—C7	1.394 (3)	C17—H17B	0.9600
C6—H6	0.9300	C17—H17C	0.9600
C7—C8	1.351 (3)	F19—C18	1.332 (2)
C7—H7	0.9300	F20—C18	1.333 (3)
C8—C13	1.426 (3)	F21—C18	1.331 (2)
C8—H8	0.9300	S22—O25	1.4276 (15)
C9—C11	1.406 (3)	S22—O24	1.4308 (14)
C9—C13	1.411 (2)	S22—C18	1.809 (2)
C9—C16	1.496 (3)	O23—S22	1.4257 (16)
			
C2—C1—C11	121.23 (19)	C9—C13—C14	119.89 (17)
C2—C1—H1	119.4	C9—C13—C8	122.18 (17)
C11—C1—H1	119.4	C14—C13—C8	117.92 (16)
C1—C2—C3	120.02 (18)	N10—C14—C5	120.50 (17)
C1—C2—H2	120.0	N10—C14—C13	120.14 (15)
C3—C2—H2	120.0	C5—C14—C13	119.36 (18)
C4—C3—C2	121.4 (2)	N10—C15—H15A	109.5
C4—C3—H3	119.3	N10—C15—H15B	109.5
C2—C3—H3	119.3	H15A—C15—H15B	109.5
C3—C4—C12	120.01 (19)	N10—C15—H15C	109.5
C3—C4—H4	120.0	H15A—C15—H15C	109.5
C12—C4—H4	120.0	H15B—C15—H15C	109.5
C6—C5—C14	119.59 (19)	C9—C16—C17	111.60 (16)
C6—C5—H5	120.2	C9—C16—H16A	109.3
C14—C5—H5	120.2	C17—C16—H16A	109.3
C5—C6—C7	121.87 (18)	C9—C16—H16B	109.3
C5—C6—H6	119.1	C17—C16—H16B	109.3
C7—C6—H6	119.1	H16A—C16—H16B	108.0
C8—C7—C6	119.9 (2)	C16—C17—H17A	109.5
C8—C7—H7	120.1	C16—C17—H17B	109.5
C6—C7—H7	120.1	H17A—C17—H17B	109.5
C7—C8—C13	121.37 (19)	C16—C17—H17C	109.5
C7—C8—H8	119.3	H17A—C17—H17C	109.5
C13—C8—H8	119.3	H17B—C17—H17C	109.5
C11—C9—C13	118.54 (16)	F21—C18—F19	106.83 (16)
C11—C9—C16	120.65 (16)	F21—C18—F20	106.57 (17)
C13—C9—C16	120.72 (17)	F19—C18—F20	107.29 (19)
C14—N10—C12	121.38 (15)	F21—C18—S22	112.10 (16)
C14—N10—C15	119.21 (14)	F19—C18—S22	112.04 (14)
C12—N10—C15	119.40 (16)	F20—C18—S22	111.67 (14)
C9—C11—C12	120.23 (15)	O23—S22—O25	116.29 (11)
C9—C11—C1	122.06 (17)	O23—S22—O24	115.01 (10)
C12—C11—C1	117.71 (18)	O25—S22—O24	114.73 (10)
N10—C12—C4	120.96 (17)	O23—S22—C18	102.68 (11)
N10—C12—C11	119.48 (17)	O25—S22—C18	102.77 (9)
C4—C12—C11	119.55 (16)	O24—S22—C18	102.49 (10)
			
C11—C1—C2—C3	1.2 (3)	C16—C9—C13—C8	−0.9 (3)
C1—C2—C3—C4	−0.5 (3)	C7—C8—C13—C9	−177.97 (18)
C2—C3—C4—C12	−1.7 (3)	C7—C8—C13—C14	1.0 (3)
C14—C5—C6—C7	0.3 (3)	C12—N10—C14—C5	−173.83 (16)
C5—C6—C7—C8	−1.1 (3)	C15—N10—C14—C5	7.4 (3)
C6—C7—C8—C13	0.4 (3)	C12—N10—C14—C13	5.7 (2)
C13—C9—C11—C12	5.4 (2)	C15—N10—C14—C13	−173.07 (16)
C16—C9—C11—C12	−178.00 (15)	C6—C5—C14—N10	−179.46 (17)
C13—C9—C11—C1	−174.00 (16)	C6—C5—C14—C13	1.0 (3)
C16—C9—C11—C1	2.6 (3)	C9—C13—C14—N10	−2.2 (3)
C2—C1—C11—C9	179.61 (16)	C8—C13—C14—N10	178.83 (16)
C2—C1—C11—C12	0.2 (3)	C9—C13—C14—C5	177.31 (17)
C14—N10—C12—C4	176.08 (16)	C8—C13—C14—C5	−1.7 (3)
C15—N10—C12—C4	−5.2 (2)	C11—C9—C16—C17	−87.9 (2)
C14—N10—C12—C11	−3.5 (2)	C13—C9—C16—C17	88.5 (2)
C15—N10—C12—C11	175.24 (16)	O23—S22—C18—F21	56.86 (17)
C3—C4—C12—N10	−176.51 (16)	O25—S22—C18—F21	−64.24 (17)
C3—C4—C12—C11	3.1 (3)	O24—S22—C18—F21	176.45 (14)
C9—C11—C12—N10	−2.2 (2)	O23—S22—C18—F19	−63.24 (18)
C1—C11—C12—N10	177.29 (15)	O25—S22—C18—F19	175.66 (15)
C9—C11—C12—C4	178.25 (16)	O24—S22—C18—F19	56.35 (18)
C1—C11—C12—C4	−2.3 (2)	O23—S22—C18—F20	176.37 (14)
C11—C9—C13—C14	−3.3 (3)	O25—S22—C18—F20	55.27 (17)
C16—C9—C13—C14	−179.86 (15)	O24—S22—C18—F20	−64.04 (16)
C11—C9—C13—C8	175.63 (15)		

### Hydrogen-bond geometry (Å, °)

**Table:**

*D*—H···*A*	*D*—H	H···*A*	*D*···*A*	*D*—H···*A*
C2—H2···O23^i^	0.93	2.47	3.369 (3)	164
C15—H15C···O24^ii^	0.96	2.40	3.276 (3)	151
C16—H16B···O25^iii^	0.97	2.58	3.377 (3)	140

Symmetry codes:  (i) *x*, *y*, *z*+1; (ii) *x*, *y*−1, *z*+1; (iii) −*x*+1, −*y*+2, −*z*+1.

### Table 2 π–π Interactions (Å, °).

**Table:**

CgI	CgJ	Cg···Cg	Dihedral angle	Interplanar distance	Offset
1	2^iv^	3.814 (2)	3.88	3.517 (2)	5.188
2	1^iv^	3.814 (2)	3.88	3.542 (2)	5.205
2	2^iv^	3.945 (2)	0.02	3.578 (2)	5.326
2	2^v^	3.874 (2)	0.02	3.440 (2)	5.181

Symmetry codes: (iv) -x, -y+1, -z+2; (v) -x+1, -y+1, -z+2.Notes: Cg1 is the centroid of ring  B (C9/N10/C11-C14), Cg2 is the centroid of ring  A (C1-C4/C11/C12). Cg···Cg is the distance between ring centroids. The dihedral angle is that between the planes of the rings CgI and CgJ. The interplanar distance is the perpendicular distance of CgI from ring J. The offset is the perpendicular distance of ring I from ring J.

### Table 3 S—O···π Interactions (Å, °).

**Table:**

X	I	J	I···J	X···J	X-I···J
S22	O23	1^vi^	3.255 (2)	3.072 (2)	146

Symmetry codes: (vi) -x+1, -y+1, -z+1.Notes: Cg1 is the centroid of ring B (C9/N10/C11-C14).
